# Physical Activity Mobile App (CareFit) for Informal Carers of People With Dementia: Protocol for a Feasibility and Adaptation Study

**DOI:** 10.2196/53727

**Published:** 2024-09-12

**Authors:** Kieren Egan, Bradley Macdonald, William Hodgson, Alison Kirk, Barbara Fawcett, Mark D Dunlop, Roma Maguire, Greg Flynn, Joshua Stott, Gill Windle

**Affiliations:** 1 Digital Health and Wellness Research Group (DHAWG) University of Strathclyde Glasgow United Kingdom; 2 Department of Physical Activity for Health University of Strathclyde Glasgow United Kingdom; 3 Department of Social Care and Social Policy University of Strathclyde Glasgow United Kingdom; 4 School of Medical and Health Sciences Bangor University Bangor United Kingdom; 5 Department of Clinical, Educational and Health Psychology University College London London United Kingdom

**Keywords:** carers, dementia, physical activity, sedentary, cross platform app, caregivers, mobile phone

## Abstract

**Background:**

Physical activity is a critical component of both well-being and preventative health, reducing the risk of both chronic mental and physical conditions and early death. Yet, there are numerous groups in society who are not able to undertake as much physical activity as they would like to. This includes informal (unpaid) carers, with the United Kingdom national survey data suggesting that 81% would like to do more physical activity on a regular basis. There is a clear need to develop innovations, including digital interventions that hold implementation potential to support regular physical activity in groups such as carers.

**Objective:**

This study aims to expand and personalize a cross-platform digital health app designed to support regular physical activity in carers of people with dementia for a period of 8 weeks and evaluate the potential for implementation.

**Methods:**

The CareFit for dementia carers study was a mixed methods co-design, development, and evaluation of a novel motivational smartphone app to support home-based regular physical activity for unpaid dementia carers. The study was planned to take place across 16 months in total (September 1, 2022, to December 31, 2023). The first phase included iterative design sprints to redesign an initial prototype for widespread use, supported through a bespoke content management system. The second phase included the release of the “CareFit” app across Scotland through invitations on the Apple and Google stores where we aimed to recruit 50 carers and up to 20 professionals to support the delivery in total. Partnerships for the work included a range of stakeholders across charities, health and social care partnerships, physical activity groups, and carers’ organizations. We explored the implementation of CareFit, guided by both Reach, Effectiveness, Adoption, Implementation, and Maintenance (RE-AIM) and the Complex Intervention Frameworks.

**Results:**

Project processes and outcomes were evaluated using mixed methods. The barriers and enablers for professional staff to signpost and use CareFit with clients were assessed through interviews or focus groups and round stakeholder meetings. The usability of CareFit was explored through qualitative interviews with carers and a system usability scale. We examined how CareFit could add value to carers by examining “in-app” data, pre-post questionnaire responses, and qualitative work, including interviews and focus groups. We also explored how CareFit could add value to the landscape of other online resources for dementia carers.

**Conclusions:**

Results from this study will contribute new knowledge including identifying (1) suitable pathways to identify and support carers through digital innovations; (2) future design of definitive studies in carer populations; and (3) an improved understanding of the Reach, Effectiveness, Adoption, Implementation, and Maintenance across a range of key stakeholders.

**International Registered Report Identifier (IRRID):**

DERR1-10.2196/53727

## Introduction

### Background

Recent estimates suggest that in the United Kingdom, 10.6 million family members and friends look after a family member or friend due to a disability, frailty, or a mental or physical condition [1]. The collective impact of this group (often termed “informal carers”) in the United Kingdom is a saving to health and social care services of £162 billion (US $206 billion, 1£=US $1.27) per year [2], a figure comparable to the entire budget for the National Health Service England [3]. However poignant this contribution is, the support does not come without personal costs to carers. Many carers report poor mental and physical health, a finding also supported by systematic review evidence [4,5]. Poor carer health is a concern for everyone in society and has been associated with a reduced quality of life and increased mortality in the person being cared for [6,7].

As global populations continue to age, our health and social care systems are beginning to reform, driven through a combination of factors (eg, staff shortages, risk of infection in hospital settings, and cost savings) to pivot further toward care in the community [8,9]. With this shift, there is a growing call to build more holistic approaches around health and well-being in carers [10,11]. Achieving such major reform looks certain to embrace digital components, including building up evidence into digital interventions that could both alleviate pressure on health and social care staff and empower carers. Yet, realizing such digital implementation remains challenging, not least because the requirements for successful implementation of digital solutions are both long and intricate [12,13]. For example, this includes not only technical and operational readiness or rigor but also the development of a strong evidence base [14], an inclusive and authentic co-design approach (ideally early and including a wide variety of key stakeholders [15]), and early recognition and management of risks around nonadoption and abandonment [16,17].

Across a wide array of carer innovations and research [18,19], the development of digital supports for carers of people with dementia continues to be of significant societal interest [20-22]. For example, the pioneering work of the World Health Organization (WHO)–led “iSupport” platform for carers of people with dementia [23] has facilitated evidence-based theories on personhood and cognitive reframing [24,25] to become accessible to a global audience through web-based training and support, with anticipated impacts including reducing burden and anxiety. The content delivered is multidimensional, including an introduction to dementia, reducing stress, supporting activities of daily living, being a carer and caring for yourself, and dealing with behavior changes. As the evidence base for iSupport continues to grow (including adaptation and evaluation in 33 countries [26]), there remains scope to complement and augment global resources such as iSupport across other unmet needs of carers. This includes areas of physical activity where the UK national survey data suggest that 81% of carers are not able to do as much physical activity as they would like to [2], with a lack of digital innovations in development for this specific group. An added challenge is that, owing to their caring role, many of the carers spend much time indoors, limiting the value of location- and step count–based approaches that are more accessible to other population groups) [27,28].

We previously reported on our initial “CareFit” progress [28,29], after building the first version of the app as an Android Package Kit (APK) and testing a minimum viable concept during the COVID-19 pandemic lockdown across Scotland. The original app was designed to support carers to undertake early steps in physical activity, informed by the UK national physical activity guidelines and is based on the transtheoretical model of behavioral change [30]. While initial results identified the acceptability, usability, and feasibility of the app and concept, the work marked only the first step to develop an evidence base for future use and explores a relatively new area of research around sedentary behavior in informal carers. We demonstrated limited implementation findings around the reach (the app was only available on Android phones), the sustainability (the app was designed for 3 weeks of use only), and the lack of wider engagement with health and social care professionals and providers. Thus, we concluded that significant insights could be gained from a redesigned version of the app (eg, a completely new build using React Native [Meta]), introducing a higher quality user experience covering multiple platforms, a more personalized user journey, and establishing how we could design a future definitive study for sustainable use with implementation. Further insights that remain critical include those relating to future study design (eg, relating to the MRC Complex Intervention Framework [15,31,32]) through the lens of a single postdiagnostic pathway such as dementia.

### Study Aims and Objectives

This work aimed to improve current uncertainties around social care pathways to implementation and gain an understanding of facilitators and barriers to the Reach, Effectiveness, Adoption, Implementation, and Maintenance (RE-AIM) [33] of “CareFit” in a real-world setting. Our objectives were to (1) expand an initial 3-week intervention to an 8-week intervention to support maintenance of physical activity; (2) develop understanding of recruitment pathways and explore barriers and enablers to recruitment for a future definitive trial, including recruiting more deprived socioeconomic groups; (3) improve understanding of usage adherence and develop understanding of the most reliable methods to regularly measure physical activity and sedentary behavior within dementia carers; (4) explore how “CareFit” could provide added value for dementia carers with the existing solution of other digital interventions (such as “iSupport”) through qualitative interviews or focus groups with key stakeholders; and (5) to explore unexpected benefits including whether people with dementia also see the benefits of CareFit.

## Methods

### Study Design

The CareFit for dementia carers study was a mixed methods evaluation of a novel motivational smartphone app to support home-based regular physical activity for unpaid dementia carers. The study took place across 16 months (September 1, 2022, to December 31, 2023) and formed a “stand-alone” element of an existing randomized controlled trial on “iSupport” [26]. Initial co-design and adaptation of an existing prototype [28] facilitated this feasibility study. In brief, the co-design sessions for adaptation and expansion involved 3 development sprints: gaining feedback from a range of stakeholders such as carers, professionals who support carers (eg, charity partners) and researchers, and developers to create priority areas for app development. At the conclusion of participation in the CareFit feasibility study (designed for up to 8 weeks of use), all carers completing their final evaluation survey received information about regional support services and a shopping voucher as a token of appreciation for their participation in this work.

### Participant Recruitment: Feasibility Study

Professional study collaborators and organizations were sent a media pack of adverts across text, image, and video formats. Informal carers of people with dementia were identified and recruited through our partner organizations’ networks, the Join Dementia Research (JDR) [34] database, and were supported by other stakeholders through advertising through posters, emails, social media, and center referrals, as required. Staff from our partner organizations were invited to contribute to the evaluation across this work, along with a broader group of stakeholders (eg, including organizations who did not take an active role in recruitment) at the conclusion of the work, for “round table” stakeholder meetings and interviews. Throughout, staff were not obliged to participate in the evaluation even where their organization was a collaborator in this work. All participants were free to withdraw at any time without any impact on their future health and care or working role. Participant data collected to the point of withdrawal were used in the analysis unless consent for this was specifically withdrawn (Textbox 1).

**Feasibility study inclusion and exclusion criteria.**

- Feasibility study inclusion criteria for carers
  - Adults (18+) living in Scotland who self-identify as an unpaid carer (partners, children, friends, etc) of a person with dementia (self-reported)
  - Contemplating or preparing to undertake physical activity
  - Ability to undertake simple exercises such as arm raises or stretching
  - Be able to read and write in English
  - Have access to a smartphone (Android or Apple) and access to the internet
  - Normal or corrected to normal eyesight
- Feasibility study inclusion criteria for social care professionals
  - Adults aged 18 and older
  - Based in Scotland
  - Working as a health and social care professional
  - Willing to engage with the study (eg, share information about “CareFit” with carers of people with dementia) through their professional role for a period of at least 3 months (to ensure timely follow-up)
- Feasibility study exclusion criteria for carers
  - Anyone advised by a clinician not to undertake physical activity or make any change in their present level of exercise
  - Are already regularly exercising to a significant level outside the home (eg, running or cycling)
  - Residing outside Scotland at the time the study is conducted
  - Currently part of a related “iSupport” study
- Feasibility study exclusion criteria for social care professionals
  - Working exclusively within the National Health Service

### CareFit Intervention

“CareFit” is a smartphone app designed to support carers of people with dementia. It was designed to be accessible on both Android and Apple smartphones and tablet devices. It was developed to support the early steps in physical activity in line with the transtheoretical model of behavior change [30] and co-designed with carers, health and social care professionals, and physical activity experts over a series of phases.

For participants who did join the study, they were sent an “invitation only” link to download the app on their own smartphone or tablet device and were supported to do this over a number of email steps and correspondence with the research team, as part of a “closed” study on Google and Apple stores.

### Ethical Considerations

#### Human Participant Ethics Review

Ethical approval for feasibility work was obtained through the Bangor University School of Medical and Health Sciences academic ethics committee (approval number 2021-16915). The initial co-design and adaptation work was supported through approval in 2022 from the Department of Computer and Information Sciences, University of Strathclyde.

#### Informed Consent

For the feasibility study, both carers and professionals were provided with online information sheets before involvement in the study. Informed consent was demonstrated by the online signing of a consent form in Qualtrics [35]. Participants electronically selected individual items in the online form, corresponding to the paper consent form, in order to confirm they had read and agreed with each item. Their electronic signature was achieved by entering their first name and surname, and then either typing or drawing their signature next to a declaration. During the consent process, potential participants were supported by a researcher in an online meeting (eg, Skype [Microsoft Corporation] and Zoom) or telephone call wherever requested and any questions were answered. They then completed an online form, or the form registered that they declined.

#### Privacy and Confidentiality

The research team and app developers undertook a number of steps to ensure the security of the information collected. While the use of secure, General Data Protection Regulation–compliant online survey forms are standard for such studies, privacy for the development and use of the app was ensured through the development of a secure content management system to store pseudonymized “top-level” information from participants about overall app use. Information that is personal to the participant, such as barriers and enablers to physical activity, was kept on the individual handset, was not shared further, and did not cross between devices, even when the user remained the same. Any information stored by the University of Strathclyde was stored within university-approved, password-protected encrypted storage sites.

#### Compensation Details

Carers who participated in this work were offered a £20 (US $25.40 [1£=US $1.27]) shopping voucher to thank them for their involvement.

### Data Collection, Management, and App Download Process

There were four key areas of data collection (1) study advertisement and recruitment impact (including proactively contacting carers through the JDR platform), (2) online surveys, (3) “In-app” data collection (namely the usage across the different domains of the app such as the “activity,” “planner,” “resources,” or “sharing” elements, including a “time stamp” at the time and date of use), and (4) interviews and focus groups. For online surveys, the online platform “Qualtrics” was used. Study adverts (in both paper-based and digital formats) were shared with study partners alongside the use of the JDR platform. Carers who consented to the study were directed to a bespoke hyperlink within the Google Play and Apple stores, where an individual pseudonymized ID was entered after download. The CareFit research team offered support to individuals at any stage as requested through email or telephone or video call support. Focus groups and interviews both took place to understand a subsample of the participants’ experiences and were used in the closing stages of the project as “round stakeholder” meetings, which explored, in conversation, future steps around key themes and points identified through the use of the RE-AIM methodology.

### Evaluation Outcomes

Our mixed methods evaluation was underpinned by a number of different data sources, including (1) baseline carer demographic survey (eg, age group, gender, and number of years caring and hours caring per week) and 8-week follow-up (eg, International Physical Activity Questionnaire Short Form, [IPAQ-SF] [36], EQ5D [37], and system usability scale [SUS] [38]), (2) professional survey (eg, elements of RE-AIM and how CareFit could integrate into working role), (3) “in-app” collected data, and (4) interviews and focus groups. Following the format of the RE-AIM framework, our outcomes were guided wherever possible by the elements of Reach, Effectiveness, Adoption, Implementation, and Maintenance and were also interpreted with support of the Complex Intervention Framework [15,31,32]. Further information is listed in Table 1.

**Table 1:** Example research questions and example data sources based on the RE-AIM framework.

RE-AIM^a^ component and example research questions		Example data sources
**Reach**		
	What is the participation rate within the study? What is the dropout rate within the study? What are the key barriers or facilitators to reach?	Registry data for “Join Dementia Research” Study monitoring of overall numbers of interviews or focus groups or roundtable discussions
**Effectiveness**		
	What are the indications around the usability of “CareFit”? What are the early indications of effectiveness for increasing physical activity/reducing sedentary behavior and increasing knowledge of physical activity? What (if any) are the unintended consequences of using the app? What is the overall caregiver engagement with the app?	Patient-reported outcome measures (eg, system usability scale, International Physical Activity Questionnaire Short Form, individual items, and bespoke measures including sedentary, knowledge, and support outcomes) Interviews or focus groups and roundtable discussions “In-app” collected data (eg, “activity,” “planner,” “resources,” or “sharing” elements including a “time stamp” at the time or date of use)
**Adoption**		
	What are the characteristics of organizations that support the work What is the organizational staff’s understanding of why CareFit was/was not adopted?	Interviews or focus group questions that focus on the experience of adoption activities
**Implementation**		
	What are the key barriers and facilitators to implementation? How can we measure the cost implications (eg, financial and time for organizations)?	Interviews or focus groups and roundtable discussions Patient-reported outcome measures (eg, EQ5D).
**Maintenance**		
	What are the facilitators and barriers to maintaining the program? Do participants continue engaging with the app across 8 weeks of use? What could encourage the use of the app beyond 8 weeks?	Interviews or focus groups and roundtable discussions. “In-app” collected data (eg, “activity,” “planner,” “resources,” or “sharing” elements including a “time stamp” at the time and date of use)

^a^RE-AIM: Reach, Effectiveness, Adoption, Implementation, and Maintenance.

### Analysis

Our approach to mixed methods [39] was to analyze qualitative and quantitative results individually to form conclusions. Where possible, we synthesized further interpretations of the quantitative results through the qualitative findings. Interviews were recorded through the use of an encrypted Dictaphone or University-approved video software where Braun and Clarke’s [40] thematic analysis was followed. For quantitative outcomes, basic statistics relating to feasibility were gathered (eg, description of key recruitment numbers, adherence to CareFit from our “in-app” data included summaries across the “activity,” “planner,” “resources,” and “sharing” elements, including a “time stamp” at the time and date of use). Indicators of usability were underpinned by SUS total scores and, on occasion, individual questions were used to support the interpretations of qualitative data. For all outcomes assessed, differences in measures between baseline and follow-up were analyzed primarily around metrics that support our understanding of feasibility and suitability of outcomes, such as completeness of information and correctness of data gathered, such as percentage completion and error value rate. Secondary analyses may be used at a later time point (eg, app engagement vs outcome improvements) for hypothesis generation but were not a primary purpose of this study.

## Results

At the time of publication (July 2024), we have recruited 41 participants into the CareFit study, with a peer-reviewed publication with the results to follow.

## Discussion

### Principal Findings

Here we aimed to expand, personalize, and evaluate the implementation potential of a cross-platform digital health app (ie, accessible on both Google and Apple stores) to support regular physical activity in carers of people with dementia for a period of 8 weeks. The results build significantly on existing research across the breadth of materials offered (eg, number and range of physical activity videos) and the new functionalities, look and feel, and interaction capabilities of the app overall. The publication of this protocol serves a number of functions to help benefit others in the field including maximizing the transparency, accountability, and reproducibility of the work, particularly given that CareFit constitutes a complex intervention [15,31,32]. For the first time, we have explored different implementation pathways and will be in a position to report on how well different routes to “marketing” for carers performed in detail—an important outcome given that carers are often difficult to identify and support in society. Furthermore, our use of both implementation focus groups guided by the RE-AIM framework provides a comprehensive understanding of how carer-focused digital health approaches (including those in dementia) can move forward at both local and national levels.

We anticipate that results will contribute new knowledge in a number of ways, including (1) recommendations of suitable pathways to identify and support carers through digital innovations; (2) future design of definitive studies in carer populations (eg, suggestions for dropout levels and suitable measures of physical activity); and (3) an improved understanding of the reach, effectiveness, adoption, and maintenance for a range of key stakeholders. Such results support the future design of this work, including the identification of an optimal and sustainable route forward for CareFit, including a pilot randomized trial for use over an extended period. This is likely to also include furthering our understanding of costs; we are currently exploring options for CareFit or future iterations to be free and as low cost as possible for carers at the point of use (July 2024).

### Limitations

There were a number of limitations of this study. First, our routes for implementation and recruitment were varied and included not only stakeholders directly advertising the study but also the JDR online registry for carers of people with dementia. Registry routes may not always reflect the wider carer demographic; however, these are increasingly recognized and used by charity partners, and it was pragmatic to use JDR within a time-limited recruitment period. Furthermore, our work identified the strengths and limitations of these different recruitment approaches. Second, there were a number of steps involved in downloading and accessing the “CareFit” app (eg, email exchanges with the CareFit research team were required to onboard participants). Such steps would not take place if the app was freely available in app stores, but we opted to design our study for “closed” testing so that we only collected app usage data from consented participants and could build up evidence in an iterative way. Finally, there were some limitations regarding our analysis of the study data. While our mixed methods approach consists of many comprehensive data sets, we opted not to analyze the personal goals, barriers, and enablers participants expressed, in order to protect their privacy. Should future research be conducted in this project or similar projects, it may be of use to expand these opportunities with the full consent of study participants as an opt in or out selection.

### Conclusions

As the role of community care in health and wellness continues to grow, new opportunities are emerging to support the role of the informal carer. Our mixed methods approach to adapting, implementing, and evaluating CareFit will not only inform the design of future similar studies but also give early indications about a wide array of impacts, such as marketing, reach, acceptability, and the feasibility of implementation.

## Abbreviations

APK: Android Package Kit
IPAQ: International Physical Activity Questionnaire
JDR: Join Dementia Research
NIHR: National Institute for Health Research
RE-AIM: Reach, Effectiveness, Adoption, Implementation, and Maintenance
SUS: system usability scale
WHO: World Health Organization

## Data Availability

The data sets generated or analyzed during this study are available from the corresponding author upon reasonable request.
